# Glia-related mechanisms in the anteroventral cochlear nucleus of the adult rat in response to unilateral conductive hearing loss

**DOI:** 10.3389/fnins.2014.00319

**Published:** 2014-10-13

**Authors:** Verónica Fuentes-Santamaría, Juan C. Alvarado, Diego F. López-Muñoz, Pedro Melgar-Rojas, María C. Gabaldón-Ull, José M. Juiz

**Affiliations:** Facultad de Medicina, Instituto de Investigación en Discapacidades, Neurológicas (IDINE), Universidad de Castilla-La ManchaAlbacete, Spain

**Keywords:** ossicle removal, microglial cells, astrocyte, cochlear nucleus, auditory pathways

## Abstract

Conductive hearing loss causes a progressive decline in cochlear activity that may result in functional and structural modifications in auditory neurons. However, whether these activity-dependent changes are accompanied by a glial response involving microglia, astrocytes, or both has not yet been fully elucidated. Accordingly, the present study was designed to determine the involvement of glial related mechanisms in the anteroventral cochlear nucleus (AVCN) of adult rats at 1, 4, 7, and 15 d after removing middle ear ossicles. Quantitative immunohistochemistry analyses at light microscopy with specific markers of microglia or astroglia along with immunocytochemistry at the electron microscopy level were used. Also, in order to test whether trophic support by neurotrophins is modulated in glial cells by auditory activity, the expression and distribution of neurotrophin-3 (NT-3) and its colocalization with microglial or astroglial markers was investigated. Diminished cochlear activity after middle ear ossicle removal leads to a significant ipsilateral increase in the mean gray levels and stained area of microglial cells but not astrocytes in the AVCN at 1 and 4 d post-lesion as compared to the contralateral side and control animals. These results suggest that microglial cells but not astrocytes may act as dynamic modulators of synaptic transmission in the cochlear nucleus immediately following unilateral hearing loss. On the other hand, NT-3 immunostaining was localized mainly in neuronal cell bodies and axons and was upregulated at 1, 4 and 7 d post-lesion. Very few glial cells expressed this neurotrophin in both control and experimental rats, suggesting that NT-3 is primarily activated in neurons and not as much in glia after limiting auditory activity in the AVCN by conductive hearing loss.

## Introduction

Conductive hearing loss is a condition that results in diminished cochlear nerve synaptic activity due to an inefficient sound transmission from the middle to the inner ear (Conlee and Parks, 1981; Tucci and Rubel, 1985). Physiological studies in adult patients and children who suffer from unilateral conductive ear disease have demonstrated that this hearing impairment results in functional anomalies in brainstem auditory evoked responses (ABRs) (Fria and Sabo, 1980; Ferguson et al., 1998). In both adult and juvenile populations, ABR recordings showed significant delays in wave V and in the III-IV interwave intervals. This observation has led to the suggestion that conductive hearing loss is associated with alterations at the central level that might have a significant impact on auditory processing. In an attempt to improve the diagnosis and treatment of these patients, animal models of conductive hearing loss have been developed to elucidate the morphological and functional anomalies associated with these pathological responses. In this regard, a series of studies have demonstrated that unilateral restriction of peripheral inputs to central auditory nuclei leads to decreased activity in auditory neurons of the affected side (Tucci et al., 1999, 2001, 2002; Hutson et al., 2007), strengthening of the ipsilateral projection from the cochlear nucleus to inferior colliculus (Moore et al., 1989), alterations in neurotransmitters release and uptake (Potashner et al., 1997; Suneja et al., 1998), redistribution of AMPA and glycine receptor subunits (Whiting et al., 2009), and modifications in the synthesis and composition of glutamate and glycine receptors (Wang et al., 2011).

Imbalance of neurotransmission after bilateral deprivation of cochlear activity results in long-term interactions between microglial cells and deafferented cochlear nucleus neurons (Campos Torres et al., 1999; Fuentes-Santamaria et al., 2012). In response to modifications in chemical and electrical signals from damaged neurons, microglia rapidly changes to an active phenotype promoting the synthesis and release of cellular mediators, as an attempt to restore synaptic homeostasis and function (Bruce-Keller, 1999; Cullheim and Thams, 2007; Hanisch and Kettenmann, 2007). Growth factors, including neurotrophins, and cytokines are activity-dependent signaling molecules involved in the regulation of synaptic activity in the healthy and injured brain (Guthrie et al., 1995; Watt and Hobbs, 2000; Hanisch, 2002; Parish et al., 2002). Studies in different lesion models such as ischemia and traumatic brain injury have shown increased production of insulin-like growth factor 1 (IGF-1) and interleukin-1β (IL-1β) by glial cells (Touzani et al., 1999; Rothwell and Luheshi, 2000; Hwang et al., 2004; Madathil et al., 2010). Particularly in the auditory system, upregulation of IGF-1 and IL-1β levels occurs in neurons but not in either astrocytes or microglia within the ventral cochlear nucleus (AVCN) of adult rats at 1, 7, and 15 d after cochlear ablation (Fuentes-Santamaria et al., 2013). These findings support the idea that additional synthesis of IGF-1 and IL-1β by glial cells is not essential to re-establish damaged auditory synaptic connections and that other molecular mediators might be involved in this process. One possible candidate is neurotrophin-3 (NT-3), a neurotrophic factor expressed in the adult and postnatal auditory system (Hafidi, 1999; Tierney et al., 2001). Apart from its role as a survival factor that, when injected into the cochlea, increases the survival of spiral ganglion neurons after deafness (Wise et al., 2011), NT-3 also participates in the reestablishment of lost synaptic connections. Supporting this concept, increases in NT-3 levels have been found in adult guinea pigs at 7d following unilateral cochlear removal, a time point at which degeneration and synaptogenesis processes take place in the cochlear nucleus (Suneja et al., 2005). It is not known, however, whether NT-3 is dynamically expressed in glial cells after auditory lesions like unilateral conductive hearing loss (UCHL).

In addition to these changes, investigations in several species have provided insights into the role that reactive astrocytes might play in restoring synaptic homeostasis after sensorineural hearing loss (Lurie and Rubel, 1994; De Waele et al., 1996; Insausti et al., 1999; Lurie and Durham, 2000; Campos-Torres et al., 2005). Particularly in the cochlear nucleus, recent findings indicate that after cochlear ablation astrocytic activation is delayed (24 h) and less persistent (<30d) relative to microglial responses, which appear earlier (16 h) and last longer (>90d) (Fuentes-Santamaria et al., 2012). These observations support the idea that although these non-neuronal elements have different temporal patterns of activation, they both are implicated in reestablishing synaptic function following deafness. In the present study, we interrupted the conduction of sound from the middle ear to the inner ear to assess whether or not glial cells express NT-3 and contribute to the recovery of synaptic deficits associated with UCHL.

## Materials and methods

### Animals

All animal protocols were approved by the Institutional Animal Care and Use Committee at the University of Castilla-La Mancha (Permit Number: PR-2013-02-03). These protocols were in accordance with the guidelines of the European Communities Council (Directive 2010/63/EU) and current national legislation (R.D. 53/2013; Law 32/2007) for the care and use of research animals. For light and confocal microscopy, 16 experimental and 4 age-matched unmanipulated control rats were used. An additional group of 12 experimental and 3 age-matched unmanipulated control rats was used for electron microscopy. Two month-old female adult rats were used for all experiments. Following the surgical procedure, the experimental animals survived for 1, 4, 7, or 15 d.

### Auditory brainstem responses (ABR)

Animals were anesthetized with isofluorane (4% for induction, 1.5–2% for maintenance with a 1 L/min O2 flow rate) and placed in a sound-attenuating electrically shielded booth (Eymasa/Incotron S.L., Barcelona, Spain) which was located inside a sound-attenuating room. Subdermal needle electrodes (Rochester Electro-Medical, Tampa, FL, USA) were placed at the vertex (positive) and under the right (negative) and left (ground) ears. The stimulation and recording were performed with a Tucker-Davis (TDT) BioSig System III (Tucker-Davis Technologies, Alachua, FL, USA). As previously reported (Alvarado et al., 2012, 2014), the ABR recordings were performed the day before the surgical procedure and at the end of each survival time. The stimuli were digitally generated using the SigGenRP software (Tucker-Davis Technologies) and the RX6 Piranha Multifunction Processor hardware (Tucker-Davis Technologies) and consisted of 5 ms rise/fall time tones, with no plateau and a cos2 envelope, delivered at 20/secat different frequencies across 7 octaves, from 0.5 to 32 kHz. They were delivered monaurally (right ear) using an EDC1 electrostatic speaker driver (Tucker-Davis Technologies) and the EC-1 electrostatic speaker (Tucker-Davis Technologies) which was placed into the external auditory meatus of the rat. Prior to the experiments, stimuli were calibrated using the SigCal software (Tucker-Davis Technologies) and the ER-10B+ low noise microphone system (Etymotic Research Inc, Elk, Groove, IL, USA). The evoked potentials were filtered (0.3–3.0 kHz), averaged (500 waveforms) and stored for later analyses on a computer. Auditory thresholds, for each of the frequencies evaluated, were determined comparing the evoked activity, recorded in 5 dB steps descending from a maximum stimulus intensity of 80 dB SPL, with the background activity measured before the stimulus onset. Auditory thresholds were defined as the stimulus intensity that evoked waves with a peak-to-peak voltage greater than 2 standard deviations above the background activity (Cediel et al., 2006; Garcia-Pino et al., 2010; Alvarado et al., 2012, 2014).

### Surgical procedure for unilateral conductive hearing loss

All surgical procedures were performed under aseptic conditions and unilaterally on the right ear. Rats were anesthetized with isofluorane as indicated above. Once the skin behind the ears was shaved, a retroauricular incision was made in order to identify the external auditory canal, which was followed to the tympanic membrane. Using fine forceps, the tympanic membrane was punctured and the malleus and incus were removed. During the surgical procedure a heating pad was used to maintain normal body temperature and recovery from anesthesia. Once awake, animals were returned to their cages and maintained with free access to food and water for the survival period.

### Primary antibodies

The antibodies used in this study are listed in Table 1. Glial and neuronal antibodies included (1) mouse anti-glial fibrillary acidic protein (GFAP); (2) rabbit anti-glial fibrillary acidic protein (GFAP); (3) ionized calcium binding adaptor molecule 1 (Iba1); (4) mouse anti-CD11b; (5) mouse anti-neuronal nuclei (NeuN) and (6) rabbit anti-neurotrophin-3 (NT-3).

**Table 1:** **Antibodies used for Immunohistochemistry**.

**Primary Antibody**	**Immunogen**	**Host**	**Code/clone**	**Dilution**	**Manufacturer**	**Tissue processing**
Iba1	C-terminus of Iba1′ (N′-PTGPPAKKAISELP-C′)	Rabbit	019–19741, Lot #CDQ5232	1:2000	Wako Pure Chemical Industries, Neuss, Germany	LM, CM, EM
CD11b	Rat peritoneal macrophages	Mouse	MCA275G	1:100	Serotec, Oxford, UK	CM
GFAP	Cow spinal cord GFAP	Rabbit	Z0334	1:2000	Dako, Glostrup, Denmark	LM, EM
GFAP	Porcine spinal cord GFAP	Mouse	MAB360	1:2000	Millipore, Billerica, MA, USA	CM
NeuN	Purified cell nuclei from mouse brain	Mouse	MAB337, Lot # LV1825845	1:200	Millipore, Billerica, MA, USA	CM
NT-3	Amino-terminal of mouse NT-3 (H-YAEHKSHRGEY-NH2)	Rabbit	AB1532SP	1:100	Millipore, Billerica, MA, USA	LM,CM

*LM, light microscopy; CM, confocal microscopy; EM, electron microscopy*.

### Immunoperoxidase staining procedure

After the appropriate post-operative survival time, control and experimental rats were anesthetized with an intraperitoneal injection of ketamine (100 mg/Kg) and xylazine (5 mg/Kg) and perfused transcardially with 0.9% saline wash followed by a fixative solution of 4% paraformadehyde in 0.1 M phosphate buffer (PB, pH 7.3). The brains were removed from the cranium, and crioprotected for 48 h. Frozen sections 40 μm thick were cut on a sliding microtome in a coronal plane. After blocking for 1 h in a solution containing 10% normal goat serum (NGS) diluted in Tris-buffered saline (TBS, pH 7.4) with 0.2% Triton X-100 (TBS-Tx 0.2%), sections were subsequently incubated overnight at 4°C in the same buffer solution with polyclonal primary antibodies for either Iba1 or GFAP or NT-3 (Table 1). The next day, sections were washed in a TBS-Tx 0.2% solution and incubated for 2 h at room temperature (RT) in biotinylated goat anti-rabbit secondary antibody (1:200, Vector Laboratories, Burlingame, CA, USA). Then, after several rinses in TBS-Tx 0.2%, sections were incubated in an avidin-biotinylated peroxidase complex (ABC) and rinsed in TBS. Sections were then exposed to 3–3′ dimanobenzidine (DAB) as the chromogenic peroxidase substrate. Care was taken to ensure that incubation times in DAB were identical across control and experimental cases. Finally, the sections were washed thoroughly, mounted on gelatin-coated slides, air-dried, dehydrated in progressive ethanol solutions, cleared in xylene, and coverslipped with Cytoseal® (Stephens Scientific, Wayne, NJ, USA). Three sets of control experiments were performed to test the specificity of immunohistochemistry detection system: (1) omission of the primary antibody by replacement with TBS-BSA; (2) omission of secondary antibodies; and (3) omission of ABC reagent. No immunostaining was detected under these conditions.

### Double immunofluorescence labeling

Sections were rinsed four times in TBS-Tx 0.2% and blocked for 1 h in the same buffer solution containing 10% NGS. Then, sections were incubated incubated overnight with one of the following mixtures of primary antibodies: (1) NeuN and Iba1; (2) NeuN and GFAP; (3) NT-3 and CD11b; and (4) NT-3 and GFAP primary antibodies. Following four 15 min rinses in TBS-Tx (0.2%), sections were incubated in the corresponding cocktail of fluorescently labeled secondary antibodies for 2 h at room temperature (1:200, anti-mouse antibodies conjugated to Alexa 594 (A-11005) and anti-rabbit antibodies conjugated to Alexa 488 (A-11008; Molecular Probes, Eugene, OR, USA) and after several rinses in TBS, they were mounted, counterstained with DAPI, coverslipped, and maintained overnight at 4°C. Sections were examined with a laser scanning confocal microscope (LSM 710; Zeiss, Germany) with excitation laser lines at 405, 488, and 594 nm, using the ZEN 2009 Light Edition software. Maximum intensity projections of a z-stack were generated. For each dye, optical sections every 2.5 μm through the thickness of the tissue were captured with a 63X Plan Apo oil-immersion objective (1.4 NA), at fixed camera gain, pinhole size, and laser intensity. Then, images were merged and saved as TIFF files.

### Electron microscopy immunocytochemistry

Animals were anesthetized as described above and perfused transcardially with 0.9% saline wash followed by a fixative perfusion of 4% paraformadehyde and 0.5% glutaraldehyde in 0.1 M PB, pH 7.3. After fixation, the brains were removed, and sectioned at 40 μm on a vibratome in the coronal plane. After several washes in PBS, sections were pre-incubated for 1 h in 10% NGS and then incubated overnight at 4°C with either Iba1 or GFAP polyclonal antibodies diluted in PBS. The following day, after several rinses in PBS, sections were incubated in a dilution of anti-rabbit secondary antibody (1:200) for 2 h at RT and after several rinses they were incubated in ABC for 1 h. Peroxidase activity was visualized with a nickel-intensified DAB reaction to produce a black reaction product. Sections containing the cochlear nucleus were post-fixed with osmium tetraoxide (1% in 0.1 M PB) for 1 h, block-stained with 1% uranyl acetate for 30 min, dehydrated in graded series of ethanol and embedded in Durcupan (Fluka) resin. Thin sections (~75 nm) in the silver-gold range were cut on an ultramicrotome (Reichert Ultracut E, Leica, Austria) and collected on 200-mesh copper grids. Tissue was observed using a Jeol-1010 electron microscope.

### Measurements of the cross-sectional area of Iba1 immunostained cells

As glial cells could modify their phenotype in response to minor changes in their cellular environment, *the cross-sectional area*, was used as an indicator of possible changes in the soma size of glial cells at the different time points after the UCHL. The cross-sectional area of Iba1 immunostained cells in both control and experimental animals was measured using the public domain image analysis software Scion Image for Windows (Scion, Frederick, MD; v beta 4.0.2). Using a 60× objective, three fields (25.16 × 10^3^ μm^2^; dorsal, middle and ventral) were sampled randomly in every fourth section throughout the rostrocaudal extent of the cochlear nucleus. Only cells with a well-defined cell body, nucleus and nucleolus were measured and included in the analysis.

### Analysis of the immunostaining

Immunostained sections from control and experimental animals were examined with bright field illumination using a Nikon Eclipse photomicroscope with a 40× objective and images captured with a DXM 1200C digital camera attached to the microscope. Color images of each field were digitized and the resultant 8-bit image contained a grayscale of pixel intensities that ranged from 0 (white) to 255 (black). As previously described (Fuentes-Santamaria et al., 2003, 2005, 2007; Alvarado et al., 2004, 2005, 2007), the densitometric procedure for the evaluation of the immunostaining was performed by using the public domain image analysis software Scion Image for Windows. Cochlear nucleus subdivisions were defined in accordance with previous terminology (for review, see Cant and Benson, 2003). The analysis of Iba1, GFAP and NT-3 immunostaining was performed in 6 coronal sections, 120 μm apart, through the rostrocaudal extent of the AVCN. In each section, three fields (55.25 × 10^3^ μm^2^ dorsal, middle and ventral) were sampled using a 40× objective. In order to perform an appropriate comparison of the immunostaining among cases, a macro was designed to process and analyze the captured images (Alvarado et al., 2004). Briefly, images were normalized by using an algorithm, based on the signal-to noise ratio that normalizes each pixel, adjusting the grayscales range of the image. Following normalization, the threshold level was set as two standard deviations above the mean gray level of the field and immunostained cells exceeding this threshold were identified as labeled. Additionally, as both the intensity and area of the immunostaining could be affected by changes in activity (Caicedo et al., 1997), *the mean gray level of the immunostaining* and *the immunostained area* were used as indicators of changes in protein levels (Winsky and Jacobowitz, 1995; Benson et al., 1997).

It has been demonstrated that the intensity of the immunostaining is related to antigen concentration (Huang et al., 1996; Yao and Godfrey, 1997). Therefore, *the mean gray level* was used as an indirect measure of intracellular protein levels within cells after UCHL providing a general estimation of the effect of the unilateral deprivation on the immunostaining of neurons and glia. Additionally, *the immunostained area* was used as an indicator of the area occupied by microglial cells and astrocytes at each survival time in comparison to control rats. It was calculated as the summed area of all profiles (cells and processes) labeled above the threshold in each field.

### Preparation of figures and statistical analysis

Photoshop (Adobe v5.5) and Canvas (Deneba v6.0) were used to adjust size, brightness and contrast of publication images. All the data were expressed as means ± standard error of the mean. Comparisons among groups were analyzed statistically using the one-factor analysis of variance and the Scheffe's *post-hoc* analysis to evaluate the effect of the survival time after unilateral conductive hearing loss over the immunostaining in the cochlear nucleus. Statistical significance was set at a level of *p* < 0.05.

## Results

### Auditory brainstem responses (ABR)

**Figure 1:**
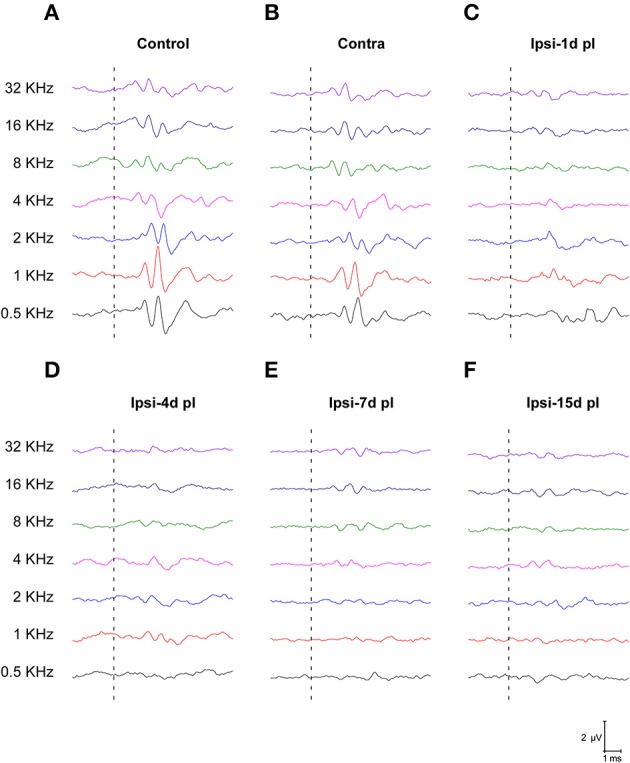
**Line graphs depicting ABR recordings at 80 dB SPL for all frequencies tested in control (A) and experimental animals following UCHL (B–F)**. In control animals **(A)** and in the contralateral side **(B)**, ABR recordings show a normal wave pattern, which was characterized by positive peaks generated following the stimulus onset (dashed lines). However, post-lesion (pl) ABRs at all timepoints, showed reduction of the responses following stimulus onset (dashed lines) at all frequencies evaluated **(C–F)**.

**Figure 2:**
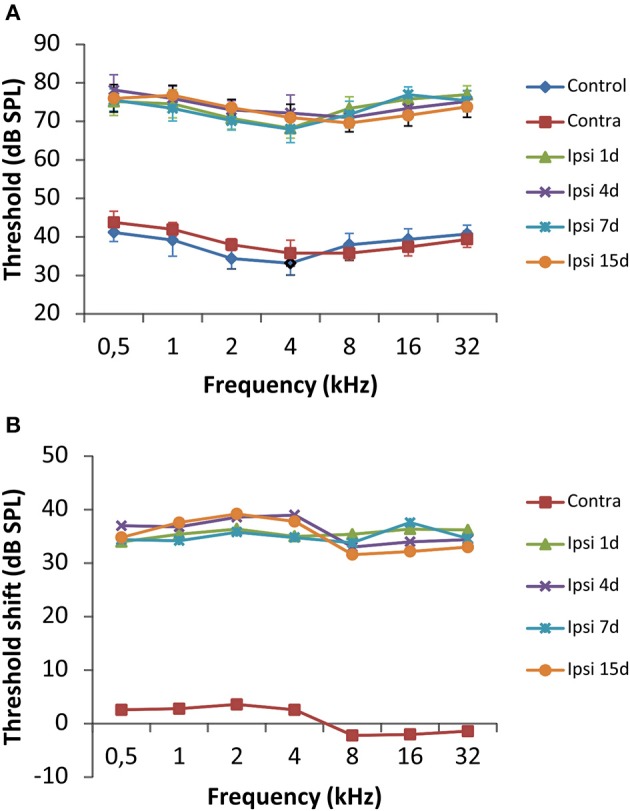
**Line graphs illustrating the effects of UCHL over the auditory thresholds at all frequencies tested**. **(A)** There was a significant threshold elevation in the ipsilateral side at all timepoints after the UCHL in comparison with control animals and the contralateral side of deprived rats. **(B)** The threshold shift was of 30–40 dB SPL in comparison to the contralateral side and unoperated rats. The error bars indicate the standard errors of the mean.

To evaluate alterations in auditory function following UCHL, ABR recordings were performed in rats before (pre-lesion ABR) and after (post-lesion ABR) unilateral ossicle removal for each of the time points described in the Materials and Methods Section. Similar to the control condition, the pre-lesion recordings showed a distinctive wave pattern characterized by four to five positive peaks generated after a stimulus (Figure 1A). Meanwhile, the post-lesion ABR in the ear ipsilateral to the lesion (Figures 1C–F) showed differences in the wave amplitudes at all the frequencies tested when compared to the contralateral side (Figure 1B) and control animals (Figure 1A). Experimental rats had significant thresholds elevations at all frequencies and time points studied after UCHL which were indicative of decreased activity in the ipsilateral auditory nuclei (Figure 2).

### Microglial response to UCHL

**Figure 3:**
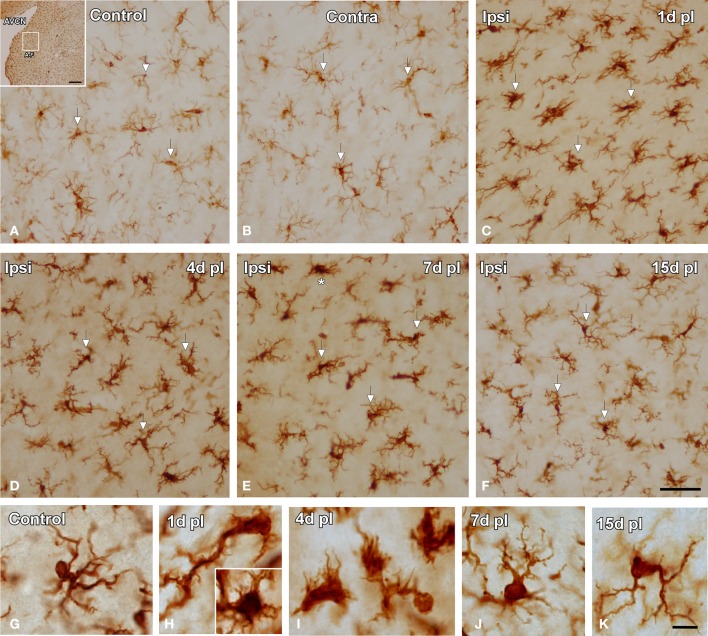
**Images depicting Iba1 immunostaining in the AVCN in control and experimental animals**. In the ipsilateral side, Iba1 immunostaining increased at 1d post-lesion (arrows in **C**) and peaked around 4d (arrows in **D**) in comparison with the contralateral side and unoperated animals (arrows in **A,B**). Iba1 levels remained elevated at 7d (arrows in **E**) and decreased at 15d (arrows in **F**) post-lesion. The morphological features of these cells are shown in **G–K**. Particularly at 7d post-lesion, activated microglial cells assumed very diverse phenotypes (compare asterisk and arrows in **E**). The inset in A indicates the location of the AVCN, and the square box indicates the approximate locations of the fields represented in **A–F**. Scale bar = 250 μm in A; 50 μm in **F**; 10 μm in K.

**Figure 4:**
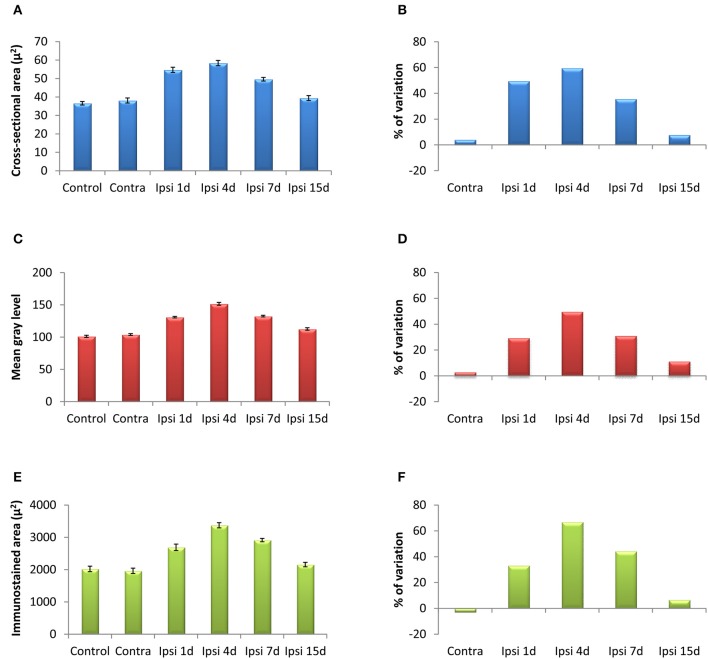
**Bar graphs showing the mean cross-sectional areas of Iba1 immunostained cells (A,B), the mean gray levels of the immunostaining (C,D) and the immunostained areas (E,F) in the AVCN of control and deprived animals**. These three indexes were significantly increased at 1, 4, and 7 d but not at 15d after ossicle removal in comparison with the contralateral side and unoperated animals. The percentages of variation for each quantitative index are shown in **B,D,F**. The error bars indicate the standard errors of the mean.

In the control condition and in the side contralateral to the lesion, microglial cells in the AVCN had round or oval cell bodies and long ramified processes (Figures 3A,B,G). A microglial reaction in the ipsilateral AVCN of experimental animals was first detectable 1 day after the lesion, at which time glial cells had larger cell bodies along with thicker and less branched processes than resting microglia (Figures 3C,H). At 4d post-lesion, microglial expression was maximal (Figures 3D,I) when compared to that in the intact side (Figure 3B) and control (Figure 3A) animals. Cells were larger, darker and occupied a larger area when compared to those observed at 1d post-lesion. These qualitative observations were confirmed by significant increases in their cross-sectional area, mean gray level of Iba1 immunostaining and immunostained area (Figure 4; Table 2). At 7d post-lesion, glial cells displayed a remarkable heterogeneity in their morphology. Some cells had smaller cell bodies and longer processes (arrowheads in Figures 3E,J) while others had morphologies resembling those seen at 1 and 4 d post-lesion (compare asterisk and arrows in Figure 3E). At this time point, the microglial cross-sectional area was significantly decreased when compared with the other survival time points. However, it was increased when compared with the unmanipulated side and control animals (Figures 3, 4; Table 2). Glial cells were still darkly immunostained as confirmed by significant decreases in the mean gray levels when compared with experimental animals at 4d post-lesion, but no differences were found when compared with 1d post-lesion animals (Figure 4; Table 2). At longer survival times (15d post-lesion), microglial cells adopted the typical ramified structure usually seen in the normal brain (Figures 3F,K, 4).

**Table 2:**
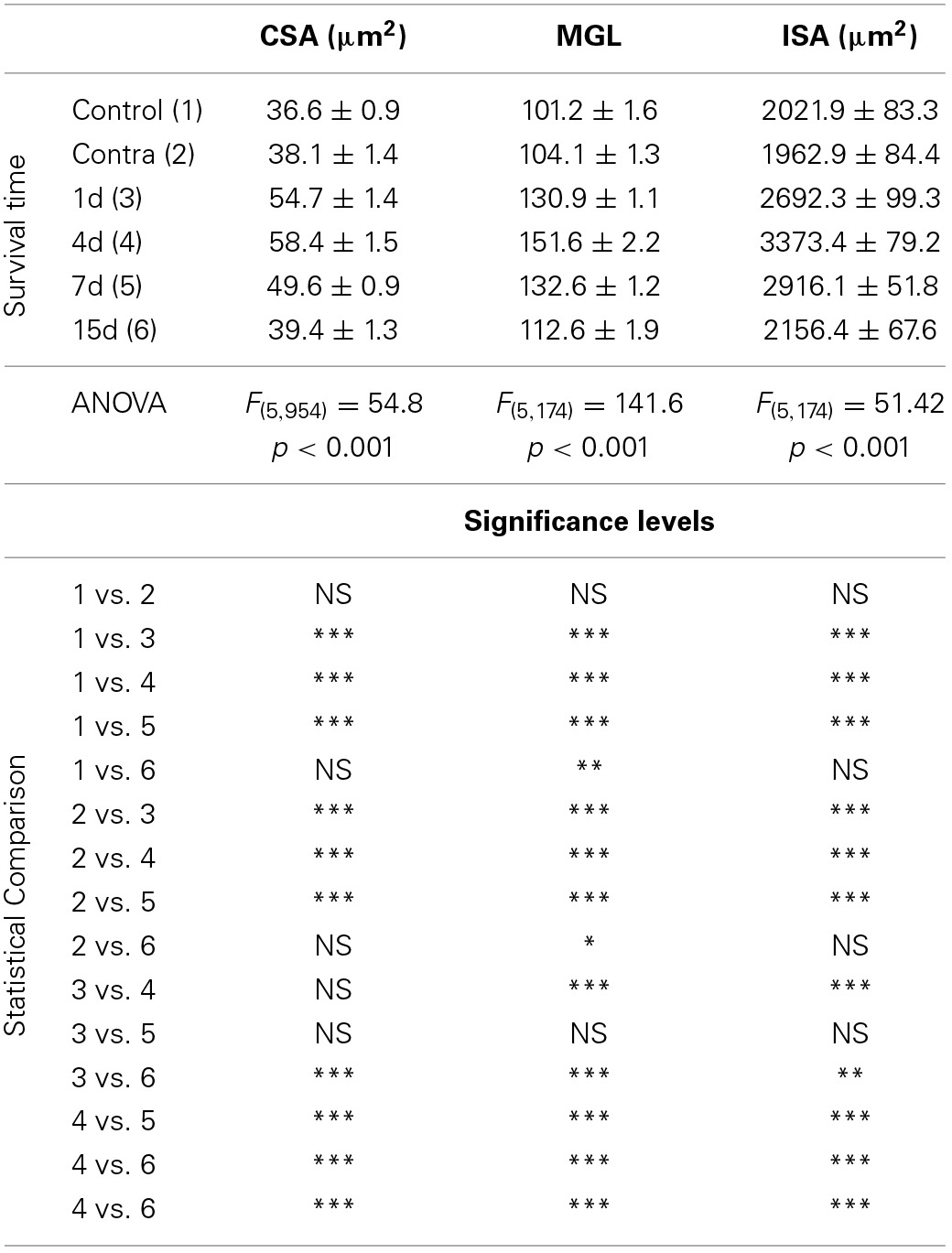
**Iba1 immunostaining in the AVCN in control and experimental animals**.

Values are means ± standard errors. CSA, Cross-sectional area of Iba1 immunostained cells; MGL, Mean gray level of Iba1 immunostaining; ISA, Immunostained area of Iba1. ^*^p < 0.05; ^**^p < 0.01; ^***^p < 0.001; NS, No significant.

**Figure 5:**
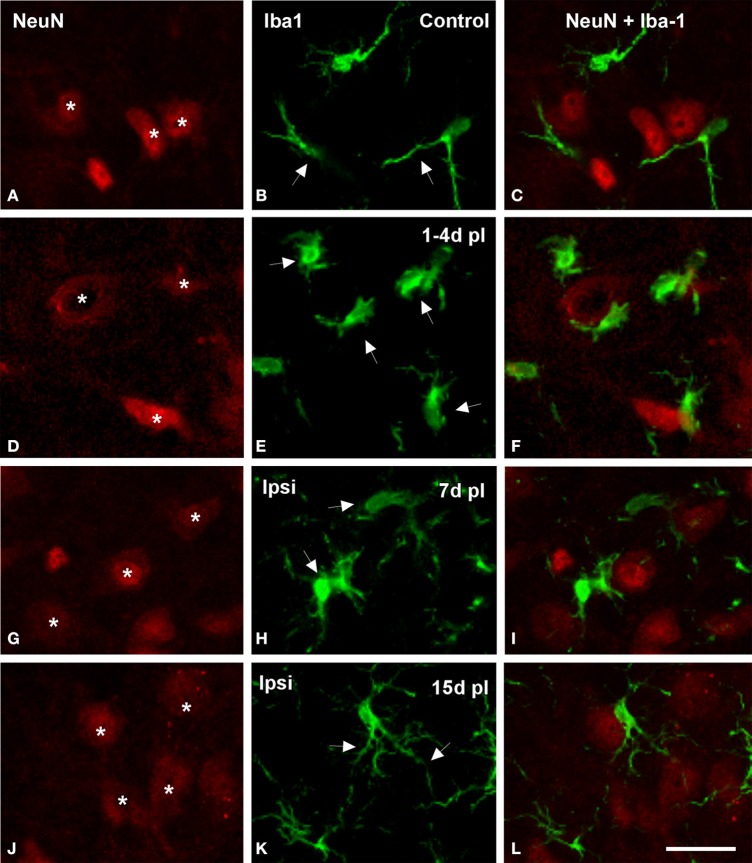
**Confocal images showing appositions between microglial cells and neurons in the ipsilateral AVCN in control and deprived rats**. In control rats, microglial cells with round or fusiform cell bodies and ramified processes were in close proximity to cochlear nucleus neurons (arrows and asterisks in **A–C**). The frequency of these appositions was particularly increased at 1 and 4 d after the lesion, when enlarged microglial cell bodies with short processes were frequently observed opposing the soma and dendrites of cochlear nucleus neurons in the affected side (arrows and asterisks in **D–F**). At later survival times after ossicle removal, the occurrence of these cellular contacts decreased (arrows and asterisks in **G–L**). Scale bar = 25 μm in **L**.

**Figure 6:**
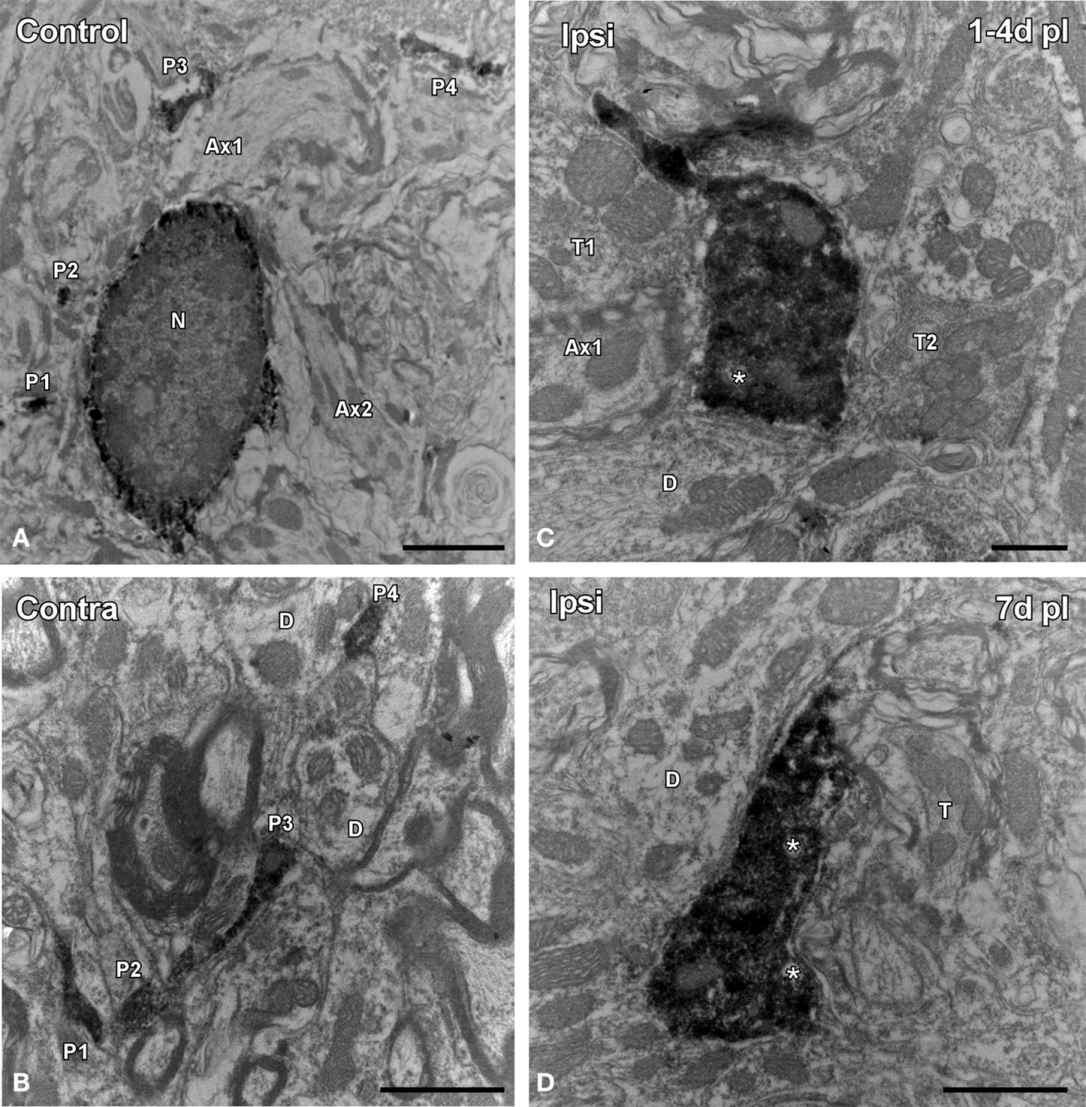
**Electron microscopy images showing the ultrastructural features of Iba1 immunostained cells in the ipsilateral AVCN in comparison to the contralateral side and control animals**. In the control condition **(A)** and in the contralateral side **(B)** to the lesion, microglial cells have a nucleus with dense heterochromatin, a cytoplasm with numerous organelles and inclusion bodies. Note that multiple labeled processes (P1–P4) of different sizes and shapes are scattered in the neuropil. Between 1 and 7 d following unilateral ossicle removal, these cells have a larger cytoplasm and thicker processes rich in vacuoles and multi-vesicular bodies (asterisks) that were seen contacting nearby synaptic elements **(C,D)**. A × 1–A × 2, axons; D, dendrite; N, nucleus; P1–P4, processes; T, terminal. Scale bar = 2 μm in **A**; 1 μm in **B,D**; 0.5 μm in **C**.

Microglial-neuronal appositions in the ipsilateral AVCN were predominantly observed at 1 and 4 d post-lesion (Figures 5D–F), when microglia assumed a more amoeboid phenotype (arrows in Figure 5E) and surrounded injured cochlear neurons (asterisks in Figure 5D). These close appositions were less frequently seen at day 7 (Figures 5G–I) and were almost absent at day 15 (Figures 5J–L) when microglial cells transformed into the ramified phenotype similar to that seen in the contralateral side and control animals (Figures 5A–C). The utrastructural features of Iba1 immunostained cells in the cochlear nucleus of control and experimental rats are shown in Figure 6. Iba1 immunostained microglial cells were identified by the presence of electron-dense DAB reaction product within their cell body cytoplasm and processes. In the control condition and the side contralateral to the lesion, microglial cells in the resting ramified state had a nucleus with dense heterochromatin packed against the nuclear membrane, a cytoplasm with numerous organelles and inclusion bodies and multiple labeled processes of different sizes and shapes in the neuropil (Shapiro et al., 2009; Figures 6A,B). In the ipsilateral AVCN of experimental animals, activated microglia was characterized by an enlarged cytoplasm and thicker processes rich in vacuoles and multi-vesicular bodies, which were observed surrounding neuronal processes and contacting nearby synaptic elements (Figures 6C,D).

### Astroglial response to UCHL

**Figure 7:**
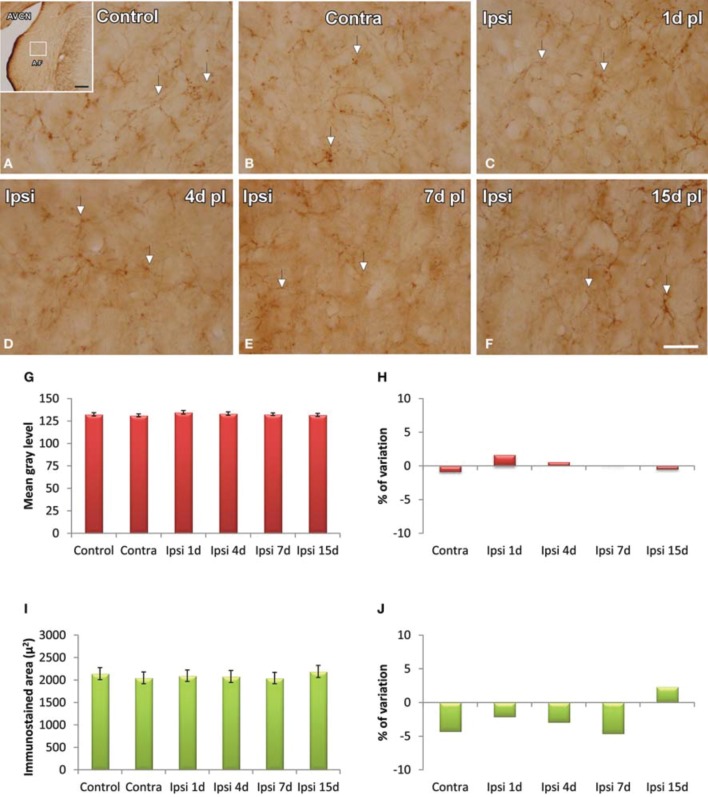
**Images depicting GFAP immunostaining in the AVCN in control rats and following unilateral ossicle removal**. GFAP immunostaining in the ipsilateral side did not change at any survival time after the lesion **(C–F)** in comparison with the contralateral side **(B)** and unoperated **(A)** animals. Bar graphs show the mean gray levels of GFAP immunostaining **(G,H)** and the immunostained areas **(I,J)**. When the two indexes were evaluated in the ipsilateral cochlear nucleus of unilaterally deprived animals at all the timepoints studied, there were no differences when compared with the contralateral side and unoperated animals. The error bars indicate the standard errors of the mean. The inset in A indicates the location of the AVCN, and the square box indicates the approximate locations of the fields represented in **A–F**. Scale bar = 250 μm in **A**; 25 μm in **F**.

**Figure 8:**
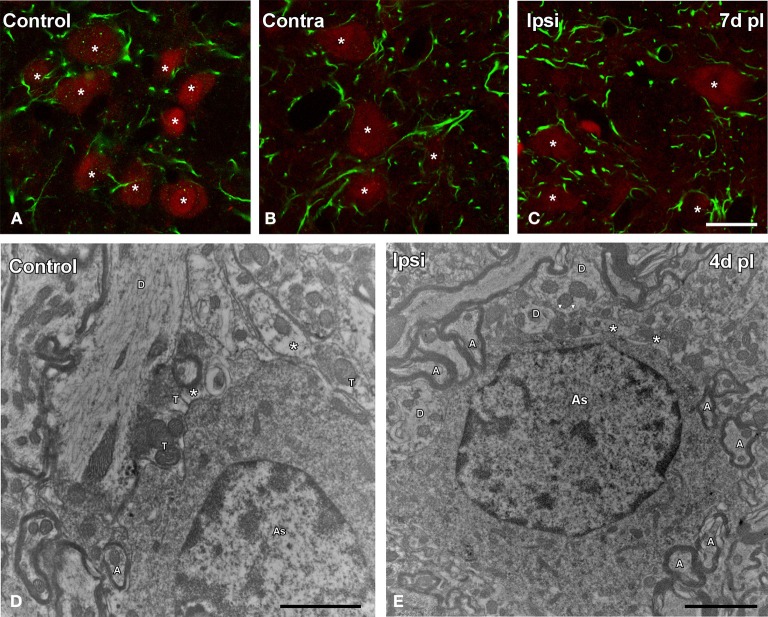
**Confocal and electron microscopy images illustrating appositions between astrocytes and cochlear nucleus neurons in control and deprived rats**. Note that astrocytic processes were distributed in the neuropil or closely associated to cochlear nucleus neurons in control and deprived animals (**A–C**). The utrastructural characteristics of these cells in the cochlear nucleus are shown in **D,E**. These macroglial cells were contacting with surrounding cellular elements of the neuropil in the control condition and after ossicle removal (asterisks in **D,E**). A, axon; As, astrocyte; T, terminal; D, dendrite. Scale bar = 20 μm in **C** (also applies to **A,B**); 1 μm in **D**; 2 μm in **E**.

GFAP immunostaining in unmanipulated animals was observed mostly as branched astroglial processes heterogeneously distributed through the AVCN (Figure 7A). Following UCHL, the morphology and staining features of astrocytes were similar to those observed in control animals (Figures 7A–F). Quantification of the mean gray levels and immunostained areas of GFAP immunostaining in experimental animals indicated that there were no differences at any survival time when compared to either the contralateral side or normal control animals (Figures 7G–J; Table 3). Similar to the control condition, astrocytic processess were found in the neuropil or closely associated with cochlear nucleus cell bodies at all the time points studied (Figures 8A–C). The utrastructural features of astrocytes in the AVCN of control and experimental rats are shown in Figures 8D,E. These macroglial cells contacted surrounding cellular elements in the neuropil (asterisks in Figures 8D,E)

**Table 3:**
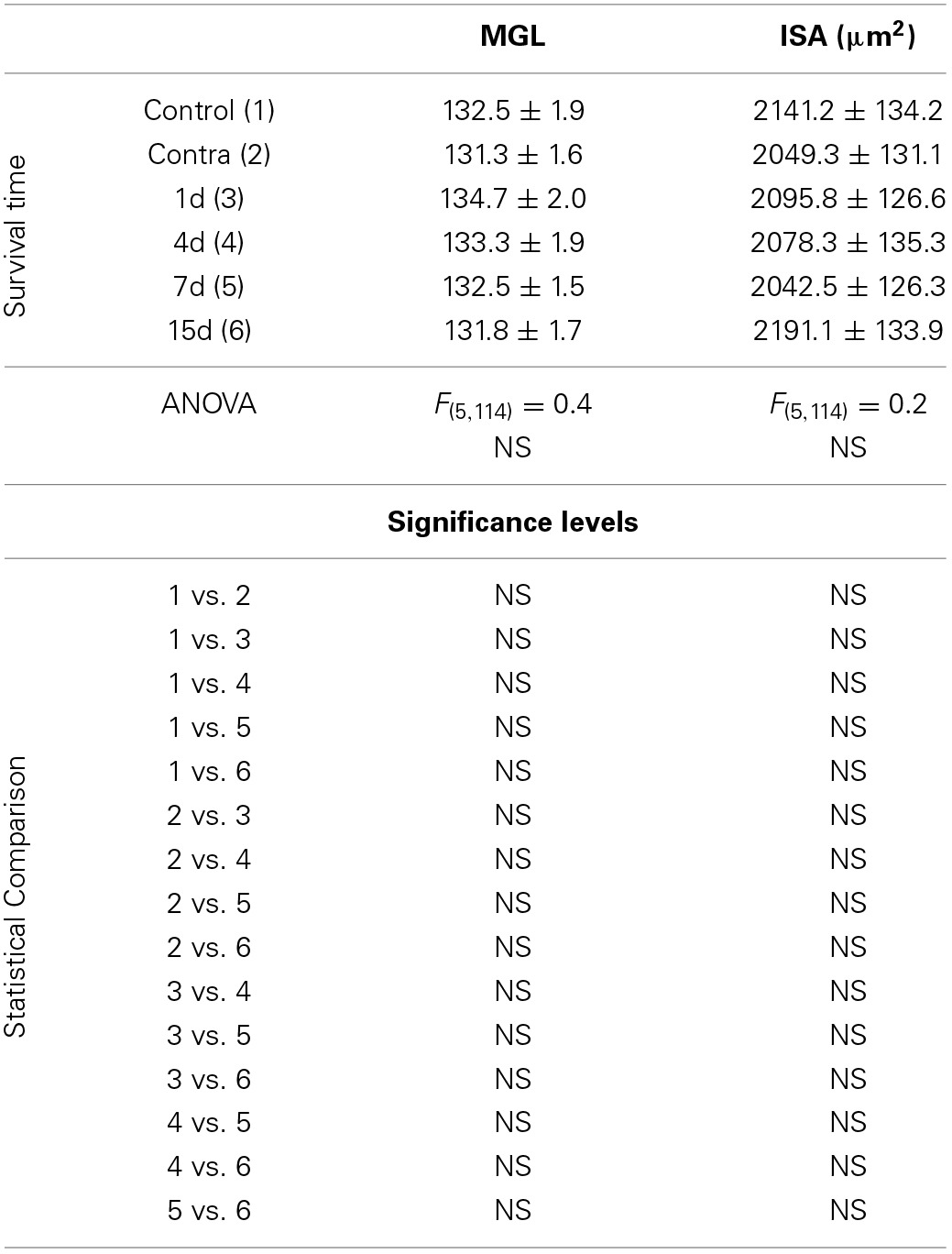
**GFAP immunostaining in the AVCN in control and experimental animals**.

*Values are means ± standard errors. MGL, Mean gray level of GFAP immunostaining; ISA, Immunostained area of GFAP; NS, No significant*.

### Upregulation of NT-3 immunostaining following UCHL

**Figure 9:**
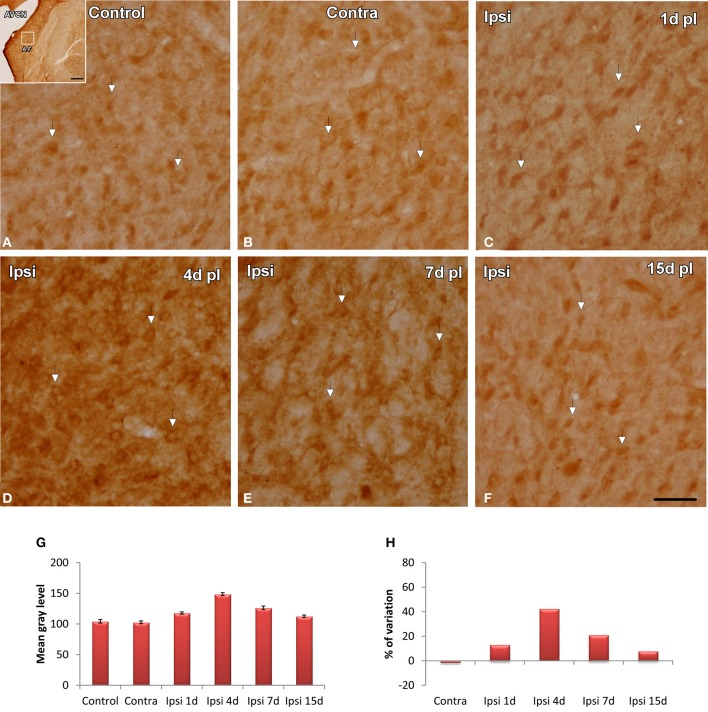
**Images depicting NT-3 immunostaining in the AVCN in control and deprived animals**. In the ipisilateral side, the immunostaining increased at 1, 4, and 7 d post-lesion (arrows in **C–E**) in comparison with the contralateral side and unoperated animals (arrows in **A,B**). Note that NT-3 levels decreased at 15d post-lesion in comparison to the other survival time points (arrows in **F**). This upregulation was confirmed by significant increases in the mean gray level of the immunostaining at the above mentioned survival times (**G,H**). The inset in A indicates the location of the AVCN, and the square box indicates the approximate locations of the fields represented in **A–F**. Scale bar = 250 μm in **A**; 50 μm in **F**.

In control and experimental animals, NT-3 immunostaining was localized within the cell cytoplasm and also in the neuropil in the AVCN (Figure 9). At 1, 4, and 7 d post-lesion (Figures 9C–E), immunostaining in the ipsilateral side was significantly increased compared to the contralateral side and to normal control animals (Figures 9A,B). These observations were corroborated by significant increases in the mean gray level of the immunostaining at the above mentioned survival times (Figures 9G,H; Table 4).

**Table 4:**
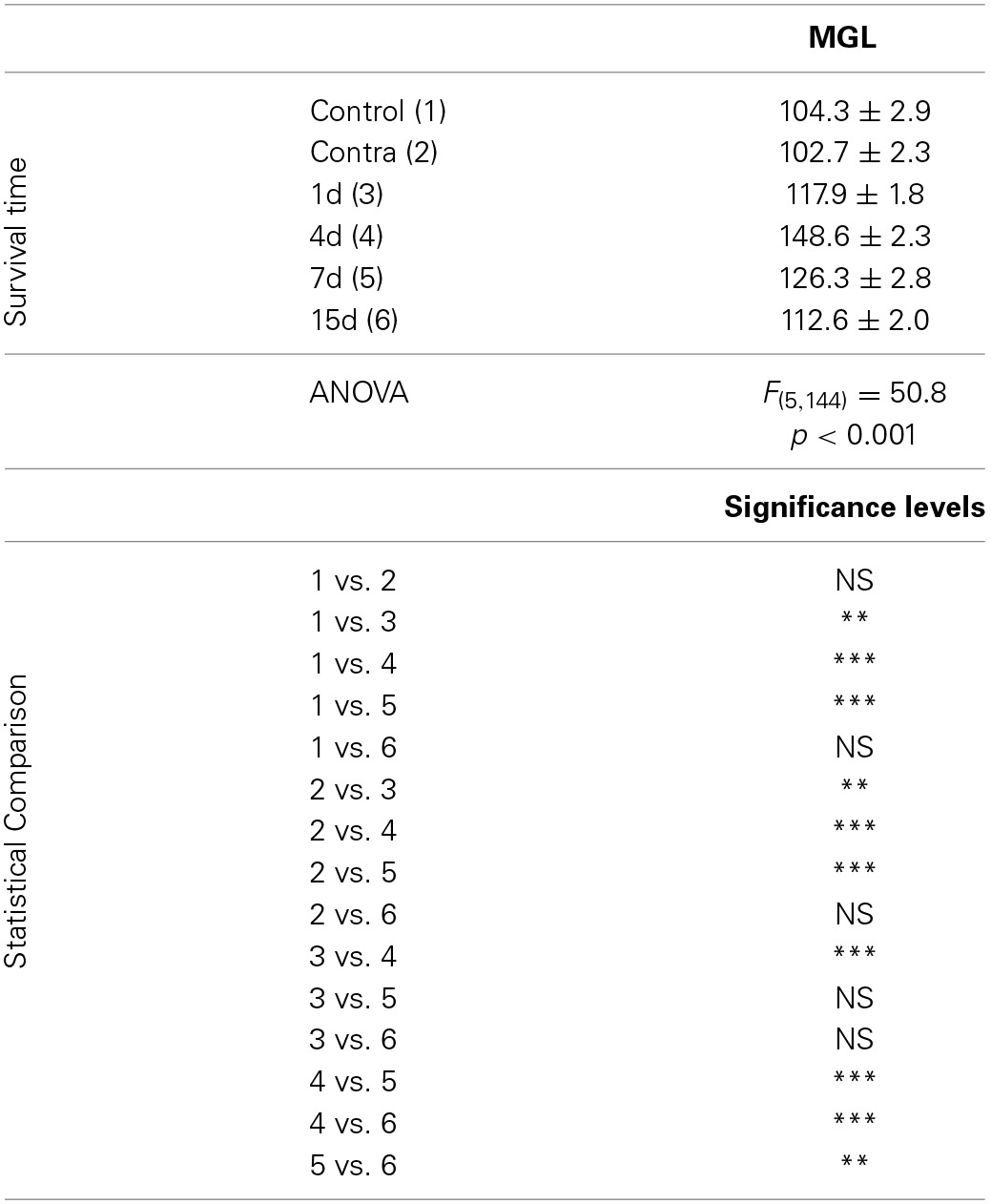
**NT3 immunostaining in the AVCN in control and experimental animals**.

Values are means ± standard errors. MGL, Mean gray level of NT3 immunostaining. ^*^p < 0.05; ^**^p < 0.01; ^***^p < 0.001; NS, No significant.

**Figure 10:**
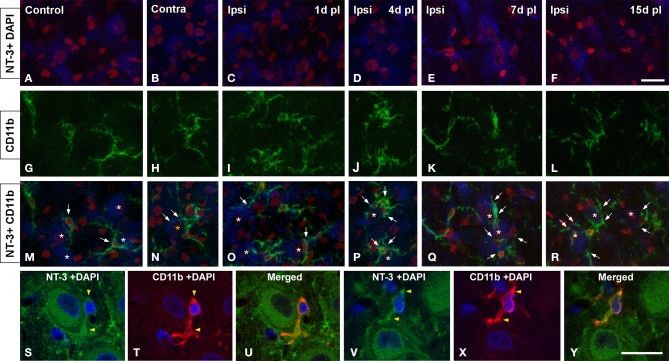
**Confocal images depicting the colocalization of NT-3 with the microglial marker CD11b in the ipsilateral cochlear nucleus for each of the time points studied after the unilateral ossicle removal in comparison to the contralateral side and control animals**. NT-3 immunostaining (blue) and cell nuclei stained with DAPI (pseudo-colored red) are shown in **A–F** while CD11b immunostained microglia (green) is illustrated in **G–L**. Appositions between microglial cells (arrows) and NT-3 stained neurons (asterisks) are indicated in **M–R** in control and experimental rats. Colocalizing cellular elements are indicated by yellow arrowheads (**S–Y**) and cell nuclei were stained with DAPI (blue). Scale bar = 20 μm in **F** (also applies to **A–R**); 20 μm in **Y** (also applies to **S–X**).

**Figure 11:**
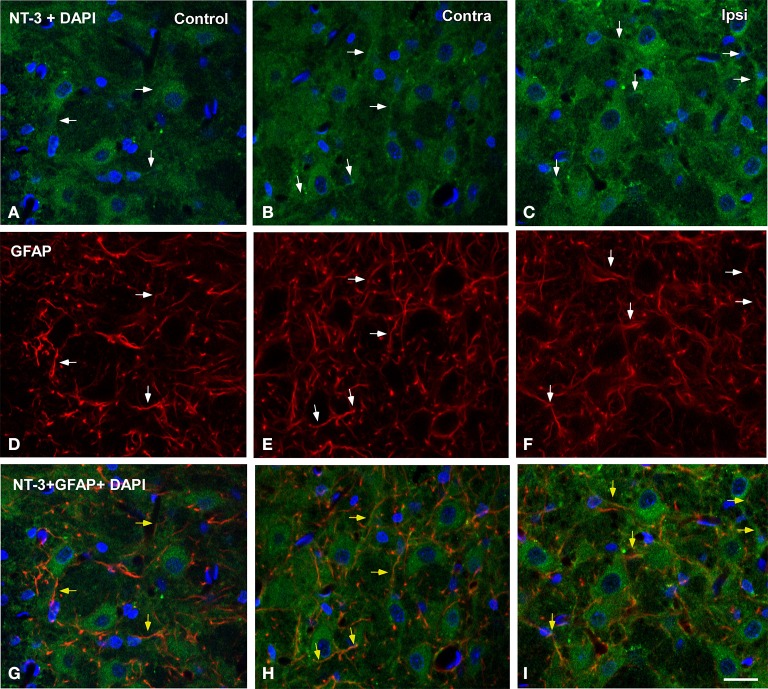
**Confocal images depicting the colocalization of NT-3 with the macroglial marker GFAP in the ipsilateral cochlear nucleus at 7d post-lesion in comparison to the contralateral side and control animals**. NT-3 immunostaining (green) and cell nuclei stained with DAPI (blue) are represented in **A–C**, while astroglial staining (red) is shown in **D–F**. Colocalization of astrocytic processes with NT-3 is shown in **G–I** (yellow arrows). Colocalyzing cellular elements in **A–F** are indicated by white arrows. Scale bar = 20 μm in **I** (also applies to **A–H**).

To determine whether glial cells expressed NT-3 following UCHL, double-labeling experiments with neuronal and glial markers were performed in both control and experimental animals. Few microglial cells (Figure 10) and astrocytes (Figure 11) colocalyzed with NT-3, demonstrating that neurons and nerve terminals are the main sources of NT-3 within the AVCN (Figures 10, 11). The colocalization of activated microglia with NT-3 in the ipsilateral side in comparison to the contralateral side and control animals is shown in Figure 10 (yellow arrowheads). Note the close spatial appositions between NT-3-containing neurons (asterisks in Figure 10) and microglial cells (white arrows in Figure 10). Regarding astrocytes, as GFAP immunostaining in the ipsilateral side was similar at all the time points studied after the lesion in comparison to the contralateral side and control animals, a representative example of the colocalization of GFAP and NT-3 in the ipsilateral AVCN at 7d post-lesion (yellow arrows) is shown in Figure 11.

## Discussion

The present study demonstrates that UCHL, which causes a progressive decline in cochlear nerve activity, leads initially to an increase in microglial but not astroglial activation in the AVCN of adult rats, at least up to 15d after the lesion. At 1 and 4 d following unilateral ossicle removal, Iba1 immunostained cells were larger and darker in the ipsilateral AVCN when compared with those in the contralateral side and control animals. These observations were confirmed by significant increases in the mean cross-sectional areas of microglial cells and increases in the mean gray levels of Iba1 immunostaining. When the expression and distribution of NT-3 and its colocalization with microglial and astroglial markers was investigated, we observed that NT-3 levels peaked by day 4 post-lesion, and that most labeling was concentrated in neuronal cell bodies and axons. Only few, scattered glial cells expressed this neurotrophin in control animals and following monoaural hearing loss. These findings suggest that microglial cells contribute to restore impaired synaptic function following UCHL.

Interrupted conduction of sound waves to the inner ear by means of unilateral middle ear ossicle removal has been reported to modify the activity of auditory neurons (Woolf et al., 1983; Tucci et al., 1999, 2001, 2002). In this regard, UCHL in gerbils results in either increases or decreases in 2-deoxyglucose (2-DG) uptake in the ipsilateral cochlear nucleus depending on whether the animals are maintained in silence following the 2-DG injection (Woolf et al., 1983) or exposed to sound (Tucci et al., 2001). Also, cytochrome oxidase activity has been shown to decrease in the ipsilateral cochlear nucleus and to increase on the contralateral side of experimental animals (Tucci et al., 2002). In agreement with these studies, our results also demonstrate decreased activity in the ipsilateral cochlear nucleus after UCHL. In this regard, increases in auditory thresholds and decrease in waves amplitudes in the ipsilateral side to the lesion were indicative of reduced levels of cochlear inputs to auditory nuclei leading to the suggestion that auditory circuitry is altered following unilateral hearing loss. Supporting this idea, increases in the number of cochlear nucleus neurons projecting ipsilaterally to the inferior colliculus of the unmanipulated side (Nordeen et al., 1983; Moore and Kitzes, 1985; Moore and Kowalchuk, 1988; Moore, 1994) and increased calcium influx in deafferented auditory brainstem nuclei that project to the inferior colliculus contralateral to the lesion (Fuentes-Santamaria et al., 2003, 2005; Alvarado et al., 2004) have been observed after experimental hearing loss in gerbils and ferrets.

The magnitude of the effects of UCHL on synaptic transmission varies depending on the species, the age of the animal when hearing loss occurs and the lesion paradigm. In the cochlear nucleus of adult guinea pigs, long-term plastic changes in GABA, glycine and aspartate uptake and release take place after middle ear ossicle removal (Potashner et al., 1997; Suneja et al., 1998). Previous studies have demonstrated that short and long-term alterations in receptor trafficking at synapses also occur in the cochlear nucleus of adult rats following monoaural earplugging (Whiting et al., 2009; Wang et al., 2011). Particularly by 1d after unilateral hearing loss, GluA3 subunits of the AMPA receptor are upregulated at auditory synapses on cochlear nucleus projection neurons while glycine receptor α1 subunits are downregulated at inhibitory synapses suggesting that decreased auditory nerve activity compromises synaptic function.

Over the years, a number of studies have demonstrated that glial cells play a pivotal role as regulators of synaptic stability in response to brain damage (Bruce-Keller, 1999; Hanisch and Kettenmann, 2007). Particularly in the cochlear nucleus, bilateral cochlear ablation triggers a microglial activation process as early as 16 h post-lesion. This response peaks at 24 h when the intracellular levels of Iba1 are increased and microglial cells have adopted a phenotype characterized by irregularly shaped hypertrophic cell bodies with very few short processes (Fuentes-Santamaria et al., 2012). The present findings indicate that reduced sound transmission to the cochlea also triggers a microglial reaction in the cochlear nucleus that is already present by 1d following the lesion and reaches maximal levels by 4d post-lesion, a time point at which activated microglia is seen in apposition to activity-deprived auditory neurons. This functional relationship has also been corroborated by electron microscopic observations demonstrating that activated microglial processes are localized in the neuropil contacting nearby cellular elements following the lesion. These ultrastructural observations are in agreement with previous studies suggesting that microglia-neuronal interactions are critical to regulate synaptic activity (Skibo et al., 2000; Shapiro et al., 2009).

One of the mechanisms used by glial cells to facilitate the exchange of cellular signals and restore synaptic homeostasis is to increase the production and release of growth factors and cytokines (Cullheim and Thams, 2007). In this regard, upregulation of IGF-1 and IL-1β levels in cochlear nucleus neurons but not in glial cells has been observed in adult rats at 1, 7, and 15 d post-ablation suggesting that deprived auditory nucleus neurons do not require additional IGF-1 and IL-1β synthesis by glial cells to re-establish affected synaptic circuits (Fuentes-Santamaria et al., 2007, 2013). Neurotrophins are also signaling molecules expressed by neurons and microglia that serve trophic roles in the normal brain (Elkabes et al., 1996; Zhang et al., 2007). In agreement with our findings, NT-3 immunostaining in rats and gerbils is localized within the cell body cytoplasm as well as in the proximal dendrites and axon hillock of cochlear nucleus neurons in the adult and developing brain (Burette et al., 1998; Hafidi, 1999; Tierney et al., 2001; Hossain et al., 2002). Interestingly, recent findings in mice have provided evidence that NT-3 might also be expressed in primary cochlear afferents which are intermingled with the principal cells of the cochlear nucleus (Feng et al., 2010, 2012). Feng et al. (2010), observed that NT-3 staining was almost absent in the cytoplasm of cochlear nucleus neurons, and hence, they hypothesized that NT-3 might be released by supporting cells and inner hair cells in the inner ear, taken by spiral ganglion neuron peripheral processes and transported anterogradely to their endings in the cochlear nucleus. It is possible that the dissimilar findings between the study of Feng et al. (2010) and the aforementioned studies in rats and gerbils together with our observations might be due to species differences or to the antibodies used by the different authors that might be recognizing different epitopes. Although in the current study we have not evaluated whether NT-3 is also expressed in synaptic endings from spiral ganglion neurons, we cannot rule out the possibility that the punctate staining observed in the neuropil and surrounding cochlear nucleus neurons is presynaptic in nature.

Neurotrophic factors are activity-dependent molecular signals that play an important role in promoting auditory nerve fiber growth and spiral ganglion neuron survival and in restoring synaptic function and structure in response to hearing loss (Suneja et al., 2005; Fukui et al., 2012). Increased neurotrophin levels have been found in the adult cochlear nucleus of guinea pigs at 7d after unilateral cochlear ablation and have been shown to contribute to synaptogenesis following cochlear damage (Suneja et al., 2005). Our results show that upregulation of neurotrophin levels within neurons occurs at 4d following UCHL, a time point at which the microglial response reached maximal levels. This upregulation, together with the fact that microglial cells did not increase NT-3 levels at any of the time points studied following the lesion, indicates that transient increases in NT-3 likely contributing to preservation of synaptic function in the cochlear nucleus after UCHL, are part of activity-modulated neurotrophic mechanisms involving cochlear neurons, but not microglial cells.

In this study we did not find evidence of astrocytes activation at any of the time points included in this study. This suggests that these glial cells may not contribute directly to neuronal and synaptic adaptations to diminished activity in the cochlear nucleus after conductive hearing loss. The ultrastructural features of astrocytes described in this study are in agreement with those presented under normal conditions in different brain structures (Aoiki, 1992; Novikov et al., 2000). Astroglial reaction has been described in the cochlear nucleus after uni- or bilateral cochleotomy (Lurie and Rubel, 1994; De Waele et al., 1996; Campos Torres et al., 1999; Insausti et al., 1999; Lurie and Durham, 2000; Fredrich et al., 2013). In the adult rat, it reaches maximal levels by 7d post-ablation when degeneration and reactive synaptogenesis are in full progress. The fact that astrogliosis seems to be more protracted and less persistent than microglial activation has led to the hypothesis that differences in the temporal pattern of activation of both cell types may reflect cooperative interactions to facilitate adult synaptogenesis following deafness (Fuentes-Santamaria et al., 2013). The fact that we do not see an astroglial reactive component, at least up to day 15 post-lesion, may complement this hypothesis by providing evidence that perhaps neuronal degeneration, like after cochleotomy, is required to unleash an astroglial reaction, whereas microglial cells react to adapt neurons and circuits to a broader range of lesion situations, ranging from attenuated activity to full-scale neuronal degeneration. Finally, although previous studies have shown that astrocytes might express NT-3 (Burette et al., 1998; Feng et al., 2012), our observations indicate that only a small subpopulation expresses this neurotrophin, both in control and experimental animals. In summary, our results provide evidence that microglial cells, but not astrocytes, are involved in a transient response to diminished activity in the cochlear nucleus, likely aimed at preserving or adapting synaptic function. Although further studies are still required, these findings suggest that modulation of the microglial responses could be a pharmacological target of interest for the treatment of pathologies that induce hearing loss.

## Author contributors

All authors had full access to all the data in the study and take responsibility for the integrity of the data and the accuracy of the data analysis. Study concept and design: Verónica Fuentes-Santamaría, Juan C. Alvarado. Acquisition of data: Verónica Fuentes-Santamaría, Juan C. Alvarado, Diego F. López-Muñoz, Pedro Melgar-Rojas and María C. Gabaldón-Ull. Statistical analysis and interpretation of data: Verónica Fuentes-Santamaría and Juan C. Alvarado. Drafting of the manuscript: Verónica Fuentes-Santamaría and Juan C. Alvarado. Critical revision of the manuscript for important intellectual content: Verónica Fuentes-Santamaría, Juan C. Alvarado, and José M. Juiz. Obtaining funding: Verónica Fuentes-Santamaría, Juan C. Alvarado, and José M. Juiz.

### Conflict of interest statement

The Associate Editor, Monica Muñoz-Lopez, and the Review Editor, Alino Martinez-Marcos, declare that, despite being affiliated to the same institution as the authors, the review process was handled objectively and no conflict of interest exists. The authors declare that the research was conducted in the absence of any commercial or financial relationships that could be construed as a potential conflict of interest.
